# P-ANCA Systemic Vasculitis Induced by Brucellosis in an Elderly Male Patient

**DOI:** 10.1155/2021/6117671

**Published:** 2021-11-25

**Authors:** Mohammed Cheikh, Abdulrahman Kabli, Esraa Sendi, Hani Almoallim

**Affiliations:** ^1^Department of Medicine, Fakeeh College for Medicine Science, Jeddah, Saudi Arabia; ^2^Department of Medicine, Faculty of Medicine, Umm Alqura University, Makkah, Saudi Arabia; ^3^Department of Medicine, Dr. Sameer Abbas Hospital, Jeddah, Saudi Arabia

## Abstract

One of the most prevalent causes of vasculitis is bacterial infection. An infection that causes anti-neutrophil cytoplasmic antibody (ANCA)-associated vasculitis (AAV) is uncommon and not reported frequently. We report a case of a 74-year-old male who presented with fever for ten days and was found to have brucellosis. Then, he was diagnosed with Guillain-Barré syndrome (GBS) and started on immunoglobulin (IVIG) for one week without a response. His fever was still persistent despite appropriate antibiotic therapy. Rheumatology evaluation revealed a history of multiple joint pain and swelling, elevated inflammatory marker, and a high titer of P-ANCA. Steroid therapy was started initially on the background of antibiotics therapy. His fever and other symptoms showed marked improvement after one week. However, P-ANCA titer was still elevated. The decision was made to treat the patient as a case of brucellosis-induced P-ANCA vasculitis. Azathioprine was added, and steroid was maintained for one month and then it was tapered gradually. All symptoms improved from the third month of follow-up except weakness from peripheral neuropathy with normalization of P-ANCA titer. His condition remained stable after six months of follow-up. Clinicians should be aware of the possibility of infection-induced vasculitis, particularly when patients' symptoms persist despite the appropriate use of antibiotics.

## 1. Introduction

Brucellosis is known to be one of the most common zoonotic infections worldwide and has a significant impact on humans, with an increased prevalence in developed countries [1, 2]. Human brucellosis is considered a life-threatening condition and has a nonspecific clinical presentation of intermittent fever, weight loss, depression, hepatomegaly, splenomegaly, and joint pain [2]. The main clinical features are fever (87%), fatigue (63%), arthralgia (62%), and muscle pain (56%) [1]. The disease is transmitted to humans from animals through ingestion of unpasteurized milk and dairy products, undercooked meat consumption, or skin penetration by contacting livestock organisms [3]. Brucellosis can present with different manifestations such as peripheral arthritis, discitis, bursitis, tenosynovitis, and osteomyelitis [4]. A systematic review reported that in the past fifteen years, there were almost 100,000 cases of brucellosis in Saudi Arabia; by progress, the number significantly started to decrease in the incidence [2].

Bacterial infection is one of the most common causes of vasculitis, and it can induce it through various direct and indirect mechanisms [5]. The pathogenesis of infections inducing vasculitis is mainly attributed to the immune response caused by the insult in most cases. Other mechanisms include a humoral immune response with immune complex formation and deposition, molecular mimicry, and cell-mediated immune response with or without granulomata formation. In contrast, immune dysregulation and anti-idiotypic response triggered by infections are not a well-known mechanism [6]. There are limited reports of infections inducing anti-neutrophil cytoplasmic antibody (ANCA)-associated vasculitis (AAV). Here, we report a case of AAV in a patient who presented with fever for ten days, who was discovered to have brucellosis. His fever and overall symptoms improved only after starting immunosuppressive therapy.

## 2. Case Presentation

A 74-year-old male patient with a known case of benign prostatic hyperplasia (BPH) presented on 17 October 2020 to the emergency department (ED) with fever and progressive generalized fatigability for 10 days. He had a history of dysuria for two weeks, in addition to polyuria, mild night sweating, and weight loss of 10 kg in one month. There was no history of upper respiratory tract infections, nausea, vomiting, diarrhea, or abdominal pain. He reported a history of drinking unpasteurized milk and contact with farm animals but denied any history of contact with tuberculosis (TB) patients. There was no history of diabetes mellitus (DM). There was no family history of malignancy or rheumatologic disease. Prior to this presentation, he had frequent visits to the ED due to increased fatigability and joint pain. However, no diagnosis was established, and the treatment was often conservative. On this admission, he was managed empirically with intravenous (IV) tazocin and IV paracetamol, given his history. A septic workup was also performed. Laboratory investigations revealed positive serology for *Brucella abortus* and *Brucella melitensis*, elevated erythrocyte sedimentation rate (ESR) (58 mm/hr), C-reactive protein (CRP) (165.08 mg/dl), and leukocytosis (23.32×10^3^/*μ*L). Chest X-ray (CXR) was unremarkable. The patient was diagnosed with brucellosis and, eventually, discharged on doxycycline and rifampin. In addition, he was scheduled for a neurological follow-up to assess his progressive fatigability.

After one month, on 17 November 2020, he was seen in the neurology clinic. The diagnosis of Guillain-Barré syndrome (GBS) was suggested as radiculoneuropathy, and axonal sensorimotor polyneuropathy was proven by electromyography (EMG) and nerve conduction study (NCS). Accordingly, the patient was admitted and received immunoglobulin (IVIG) as recommended by the neurologist. After one week of receiving IVIG, his condition was still not improving and he was referred to the outpatient rheumatology clinic to exclude possible vasculitic processes. Later on, the patient was seen in the rheumatology clinic complaining of multiple joint pain and swelling of wrists, fingers, elbows, knees, ankle, and foot, which started four weeks earlier to his visit.

The pain and swelling in the knees and elbows almost completely improved after brucellosis therapy. However, he was still complaining of pain and swelling in the other joints, which usually worsened at night and improved with movement. Perinuclear anti-neutrophil cytoplasmic antibodies (p-ANCA) was strongly positive (131), ESR was 53 mm/1 hr, and CRP was 132.83 mg/dL. Rheumatoid factor (RF), anti-citrullinated protein antibodies (ACPA), antinuclear antibodies (ANA), cytoplasmic anti-neutrophil cytoplasmic antibody (C-ANCA), and ferritin were all normal. Despite being on appropriate antibiotics treatment for brucellosis, his fever did not subside. It was recurring in episodes, with each episode lasting 20 minutes per day. The fever was not associated with sweating, shivering, or other constitutional symptoms. Furthermore, based on the constellation of several findings, which include the persisting fever despite appropriate antibiotic therapy, the EMG/NCS finding of axonal sensorimotor polyneuropathy, the persistence of elevated inflammatory markers, the positive P-ANCA, and imaging that did not show any masses or fluid collection, the likelihood of underlying systemic vasculitis was high according to the rheumatology team. Therefore, prednisolone 50 mg PO OD was started. In addition, he was continued on doxycycline and rifampin for another set of 11 days to complete six weeks of the brucellosis treatment course.

After one week of being on steroids, his fever totally subsided and other symptoms had significantly improved. Also, his CRP started to decrease, reaching 79.59 mg/dl. However, P-ANCA was still high (119.4). Therefore, the decision was made to continue on prednisolone 50 mg for another week, followed by 40 mg for two weeks and then 30 mg for two weeks. On the next visit after four weeks on 27 December 2020, he reported significant improvement in his symptoms with no more joint pain and resumed his normal activities independently. Also, lab results showed decreased P-ANCA to 57.8 and CRP to 19.36 mg/dL. Azathioprine 50 mg PO twice a day was added while he continued taking steroids with a tapering dose. After four weeks, on 26 January 2021, he reported that he was no longer experiencing fever, fatigue, joint swelling, or pain. However, he could not actively flex or extend his left index finger or right thumb, which was attributed to his peripheral neuropathy. Therefore, azathioprine dose was optimized to 50 mg three times a day. The patient was reevaluated on 7 March 2021. He was found to be asymptomatic, with no active complaints. Subsequently, azathioprine dosage was reduced to 50 mg PO BID.

## 3. Discussion

We report a case of brucellosis-induced P-ANCA vasculitis in a patient with a history of fever, fatigue, polyarthritis, and findings of peripheral neuropathy. The diagnosis of brucellosis was confirmed based on clinical presentation and biochemical evidence with positive *Brucella* antibodies. In this case, the multisystem involvement could be justified by chronic indolent infections such as brucellosis. However, the patient received antibiotics to treat brucellosis, but his fever persisted with a lack of response. Furthermore, specific clinical findings, such as polyarthritis and peripheral neuropathy, were not explained by underlying mechanisms, raising suspicion of systemic autoimmune processes. The patient's condition has not improved after IVIG therapy. The patient was put on steroids at a rheumatology clinic and showed substantial improvement from the commencement of the drug till he was almost asymptomatic with no active complaint. The possibility of underlying autoimmune processes such as vasculitis, which might be triggered by infection, was proposed.

Infectious agents can cause vasculitis through various mechanisms, which can be divided into two main categories: direct and indirect. In the former, infectious pathogens directly damage the vascular wall, triggering an inflammatory reaction [7]. They trigger an immunological response against blood vessels in the latter, the indirect approach. Because they can share epitopes with the host or change self-antigens, causing an immune system cross reaction, this mechanism results in immunological reactions. However, distinguishing between direct and indirect types of vasculitis is challenging since most infectious agents may induce vasculitis in both ways.

The initiating events of AAV are not well understood. Several factors may be responsible, e.g., genetic factors, infectious pathogens, drugs, environmental exposure, and others. Research efforts have focused on the identification of pathogens that may precipitate vasculitis [8]. As the clinical presentations of AAV may overlap with many infectious agents, it is essential to identify pathogens with the ability to precipitate AAV. This association between getting an infection and the development of autoimmune disease afterward is well-recognized. This could be explained by molecular mimicry, e.g., autoantibodies to lysosomal membrane protein-2 (LAMP-2); a new ANCA subtype has been identified that is associated with ANCA-associated glomerulonephritis. This further commonly recognizes the human LAMP-2 epitope (designated P (41–49)) with 100% homology toward the bacterial adhesion type 1 fimbrin D-mannose specific adhesion (FIMH), in which they cross react [9]. Links with infection via homology, e.g., the exposure to *S. aureus*, may induce anti-complementary proteinase 3 (PR3) antibodies that, in turn, induce anti-PR3 antibodies or C-ANCA via an anti-idiotypic response and AAV [10]. It is not entirely understood how brucellosis in the case presented might have triggered this extensive vasculitic reaction.

Several factors support the presence of an underlying autoimmune disease process in our patient including insufficient clinical improvement at the beginning despite being on the appropriate antibiotics for brucellosis treatment, persistently high inflammatory markers, strongly positive P-ANCA antibody, the pattern of polyarthritis that did not usually associate with brucellosis, and polyneuropathy that did not respond to IVIG. The observation of marked improvement on steroid therapy had further supported the underlining vasculitic process. There was a significant improvement in inflammatory markers and the titer of P-ANCA with treatment. We encourage early involvement of rheumatology service whenever there is a complex disease presentation and inappropriate response to a standard therapy like in our case. This is to avoid delay in diagnosing severe complications from common infections.

There are typical clinical manifestations of brucellosis. It is one of the rare bacterial infections with musculoskeletal (MSK) symptoms, usually affecting the sacroiliac joint in up to 80% of individuals with osteoarticular illness, the spinal joint (up to 54%), and, less frequently, the peripheral joints [4]. Surprisingly, the peripheral joints most frequently implicated with brucellosis are the knees, hips, and ankles [4]. In our case, the patient did not have any sacroiliac or spine involvement. His primary arthritis, which did not react to antibiotic treatment, was in his small joints of the hands and feet. This probably suggested a separate disease like vasculitis driving this type of polyarthritis.

There are many reported cases in the literature linking *Brucella* infection with vasculitis. Vicario et al. [11] reported a case of a 29-year-old female with brucellosis presenting with granulomatous vasculitis. Dizbay et al. [12] described a case of brucellosis complicated with renal failure and leukocytoclastic vasculitis, in which their case had positive P-ANCA, RF, hypocomplementemia, and increased levels of polyclonal immunoglobulins (IgG, IgA, and IgE) and they thought that mixed cryoglobulinemia was the cause of this presentation. Similarly, our patient developed vasculitis with a positive P-ANCA following his infection with *Brucella*, but we did not identify any hypocomplementemia or increased levels of polyclonal immunoglobulins. Korkmaz et al. [13] and Nagore et al. [14] reported cases of brucellosis presenting with leukocytoclastic vasculitis findings that were not present in our case. These reports indicate a potential underlying immunological dysfunction linked with brucellosis including P-ANCA vasculitis.

Brucellosis is a disease that can affect the central and peripheral nervous system, and it has variable neurological manifestations. However, brucellosis infection that presents with acute peripheral neuropathy mimicking GBS-like manifestations was reported among 19 cases according to Alanazi et al. [15] Although our patient had confirmed signs of peripheral neuropathy, his presentation lacks any signs of meningitis, encephalitis, brain abscess, myelitis, or radiculitis, commonly seen in neurobrucellosis. It is recommended to investigate for *Brucella* among patients in endemic regions presenting with acute peripheral neuropathy [15]. On the other hand, peripheral neuropathy is a frequent finding in AAV and may occur in up to 70% of patients with microscopic polyangiitis and 15% of patients with granulomatosis and polyangiitis [16]. Clearly, peripheral neuropathy in our patient was most likely related to induced vasculitis. Therefore, with atypical presentations of brucellosis, autoimmune disease process should be considered. The delay in obtaining a rheumatology consultation may explain why our patient was treated with IVIG which probably was not indicated in his case. This emphasizes that patients with joint problems may not receive appropriate evaluation especially when they are part of a systemic disease like in our case.

In conclusion, the lack of clinical response to the standard course of antibiotics, presence of persistent fever, peripheral neuropathy, elevation for CRP, and strongly positive P-ANCA increased the likelihood of brucellosis-induced P-ANCA vasculitis in this elderly male patient. There was an excellent response to steroid and azathioprine in association with a full six-week course of antibiotics treatment for brucellosis. We ought to increase the physician's awareness of the presence of atypical presentations of AAV triggered by acute brucellosis.

## Data Availability

Data can be obtained from the corresponding author upon request.
